# 11β hydroxysteroid dehydrogenase 1: a new marker for predicting response to immune-checkpoint blockade therapy in non-small-cell lung carcinoma

**DOI:** 10.1038/s41416-020-0837-3

**Published:** 2020-04-27

**Authors:** Ryoko Saito, Yasuhiro Miki, Takuto Abe, Eisaku Miyauchi, Jiro Abe, Ren Nanamiya, Chihiro Inoue, Ikuro Sato, Hironobu Sasano

**Affiliations:** 1 Department of Pathology, Tohoku University School of Medicine, Miyagi, Japan; 2 Department of Respiratory Medicine, Tohoku University Hospital, Miyagi, Japan; 3 Department of Thoracic Surgery, Miyagi Cancer Center, Miyagi, Japan; 4 Department of Pathology, Miyagi Cancer Center, Miyagi, Japan

**Keywords:** Non-small-cell lung cancer, Predictive markers

## Abstract

**Background:**

Understanding the status of intratumoural immune microenvironment is necessary to ensure the efficacy of immune-checkpoint (IC) blockade therapy. Cortisol plays pivotal roles in glucocorticoid interactions in the immune system. We examined the correlation between intratumourally synthesised cortisol through 11β hydroxysteroid dehydrogenase (HSD) 1 and the immune microenvironment in non-small-cell lung carcinoma (NSCLC).

**Methods:**

We correlated 11βHSD1 immunoreactivity in 125 cases of NSCLC with the amount of intratumoural immune cells present, and 11βHSD1 immunoreactivity with the efficacy of IC blockade therapy in 18 specimens of NSCLC patients. In vitro studies were performed to validate the immunohistochemical examination.

**Results:**

11βHSD1 immunoreactivity showed a significant inverse correlation with the number of tumour-infiltrating lymphocytes and CD3- or CD8-positive T cells. 11βHSD1 immunoreactivity tended to be inversely correlated with the clinical efficacy of the IC blockade therapy. In vitro studies revealed that 11βHSD1 promoted the intratumoural synthesis of cortisol. This resulted in a decrease in cytokines and in the inhibition of monocyte migration.

**Conclusions:**

Our study is the first report clarifying the inhibitory effects of intratumourally synthesised cortisol through 11βHSD1 on immune cell migration. We propose that the response to IC blockade therapy in NSCLC may be predicted by 11βHSD1.

## Background

Immune-checkpoint (IC) blockade therapy has been used for patients with various malignant tumours, including non-small-cell lung carcinoma (NSCLC), and has contributed to substantial improvements in patient prognoses. In the field of NSCLC, this therapy is used alone or in combination with chemotherapy as first-line treatment. The immunohistochemical expression of programmed death-1 (PD-1)–programmed death-ligand 1 (PD-L1) in tumour cells is used as a predictor of a patient’s response to a single therapy of IC blockade.^1^ However, an adequate response can be observed in only 30.3–39.3% of NSCLC, in spite of the high expressions of PD-L1.^2^ Therefore, it is necessary to find new predictors or to provide new strategies using this therapy.

We focused on glucocorticoid (GC), especially intratumourally synthesised cortisol (active GC) as a factor. GC has been well-known as one of the dominant factors influencing the intratumoural immune microenvironment, especially inhibiting the migration of immune cells. In addition, the direct contact of tumour-infiltrating lymphocytes (TILs), including CD8-positive T cells, with tumour cells has been reported to modulate the effects of IC blockade therapy through the tumour’s suppressive effects.^3–5^

After binding of the GC to the GC receptor (GR), the GC–GR complex subsequently translocates into the nucleus and binds to the GR-responsive element (GRE). This complex upregulates immunosuppressive genes such as IκB and interleukin (IL)-10 by binding to the positive GRE or downregulates inflammatory genes such as IL-6, IL-8, chemokine (C–C motif) ligand 5 (CCL5) and tumour necrosis factor α by binding to the negative GRE mainly in the genomic mode.^6–8^ In addition, the non-genomic mode induced rapid GC responses, resulting in transmembrane currents, phosphorylation and calcium mobilisations.^6^ The effects of GC differ in a tissue-dependent manner.^6,8,9^ GC was also reported to inhibit the proliferation of breast and lung carcinoma cells.^10,11^ The level of GR immunoreactivity is significantly correlated with a good prognosis in NSCLC.^12^ There were studies that reported that GC increased the invasion of epidermal keratinocyte cell lines through GR and reduced the sensitivity to chemotherapy in NSCLC.^11,13^ However, the effects of GC on the intratumoural immune microenvironment, including TILs, have not been well clarified in human malignancies.

Cortisol is synthesised mainly in the adrenal gland but also in other organs. In these organs, cortisol, a biologically active GC, is converted from cortisone, a biologically inactive GC, by 11β hydroxysteroid dehydrogenase (HSD) 1. The reverse conversion process from cortisol to cortisone is regulated by 11βHSD2. Therefore, the presence of 11βHSD1 could increase in situ concentrations of cortisol available for GR.^9^ Thus, its increased expression in situ could also enhance the actions of GC in its target tissues. In addition, the local balance between these two enzymes regulates GC actions. It is important to evaluate the status of both 11βHSD1 and 11βHSD2 in situ in order to further explore GC actions in GR-positive target tissues. Lung cancer itself was reported to express both of these enzymes with higher 11βHSD2 in adenocarcinoma than in squamous cell carcinoma.^13–15^ However, the status of 11βHSD1 and its role in immune system and cancer progression of NSCLC has remained virtually unknown.

In this study, we initially evaluated the correlation between the status of 11βHSD1 and the tissue- immune microenvironment in NSCLC. This evaluation included its correlation with the efficacy of IC blockade therapy, and the effects of cortisol on the production of cytokines in NSCLC cells. Our goal was to identify new predictors of the efficacy of IC blockade therapy and provide new insights into the therapeutic strategies of NSCLC.

## Methods

### Lung cancer cases

We examined a total of 125 NSCLC cases who were all Japanese and underwent surgical resection from 2014 to 2015 at the Department of Thoracic Surgery, Miyagi Cancer Center, Miyagi, Japan. All these patients had not received chemotherapy or irradiation prior to surgery. The cases included 95 adenocarcinoma (53 males and 42 females, median age: 68.0 years, range: 41–82 years and standard deviation: 9.3 years) and 30 squamous cell carcinomas (27 males and 3 females, median age: 72.0 years, range: 56–83 years and standard deviation: 6.4 years). We examined the sections that include the largest diameter or the most representative sections of each tumour. Apart from this cohort, as a new cohort, we also studied 18 cases in order to explore the correlation between 11βHSD1 immunoreactivity and the therapeutic efficacy of IC blockade therapy, in the biopsy or surgical specimens of NSCLC patients retrieved from 2017 to 2019 at Tohoku University Hospital, Miyagi, Japan. These 18 cases did not harbour any mutations of epidermal growth factor receptor and anaplastic lymphoma kinase, and demonstrated relatively abundant PD-L1 immunoreactivity assessed as high expression (the total proportion score: >50%) according to KEYNOTE-024 (ClinicalTrials. gov, NCT02142738). All 18 cases were treated with pembrolizumab following the pathological diagnosis. The clinical therapeutic efficacy was assessed according to the Response Evaluation Criteria in Solid Tumours (RECIST) version 1.1.^16^ There were no cases showing pseudoprogression after the pembrolizumab treatment. All the specimens were fixed in 10% formalin and embedded in paraffin. The study protocol was approved by the Ethics Committee at the Tohoku University School of Medicine and Miyagi Cancer Center, respectively.

### Immunostaining

The characteristics of the antibodies used were as follows: 11βHSD1 ([Abcam, Cambridge, UK], EP9406(2), Rabbit monoclonal, 1:200, Autoclave in citrate buffer), 11βHSD2 ([Santa Cruz Biotechnology, Santa Cruz, USA], C-9, Mouse monoclonal, 1:200), GR ([Cell Signaling Technology, Danvers, MA, USA], D6H2L, Rabbit monoclonal, 1:400, Autoclave in citrate buffer), CD3 ([Dako, Glostrup, Denmark], F7.2.38, Mouse monoclonal, 1:500, Autoclave in citrate buffer) and CD8 ([Dako, Glostrup, Denmark], C8/144B, Mouse monoclonal, 1:50, Autoclave in citrate buffer). Immunohistochemistry for PD-L1 was performed using the Dako PD-L1 22C3 pharmDx kit (Dako, Carpinteria, CA) on the Dako Link 48 platform. A Histofine Kit (Nichirei, Tokyo, Japan) using the streptavidin–biotin amplification method was used for 11βHSD1, GR, CD3 and CD8, and the EnVision kit (Dako, Agilent Technologies, Inc., Santa Clara, CA, USA) was used for 11βHSD2 in this examination. The antigen–antibody complex was visualised using the 3,3ʹ-diaminobenzidine (DAB) solution (1 mM DAB, 50 mM Tris-HCL buffer, pH 7.6 and 0.006% H_2_O_2_) and counterstained with haematoxylin.

### Evaluation of immunoreactivity

**Fig. 1:**
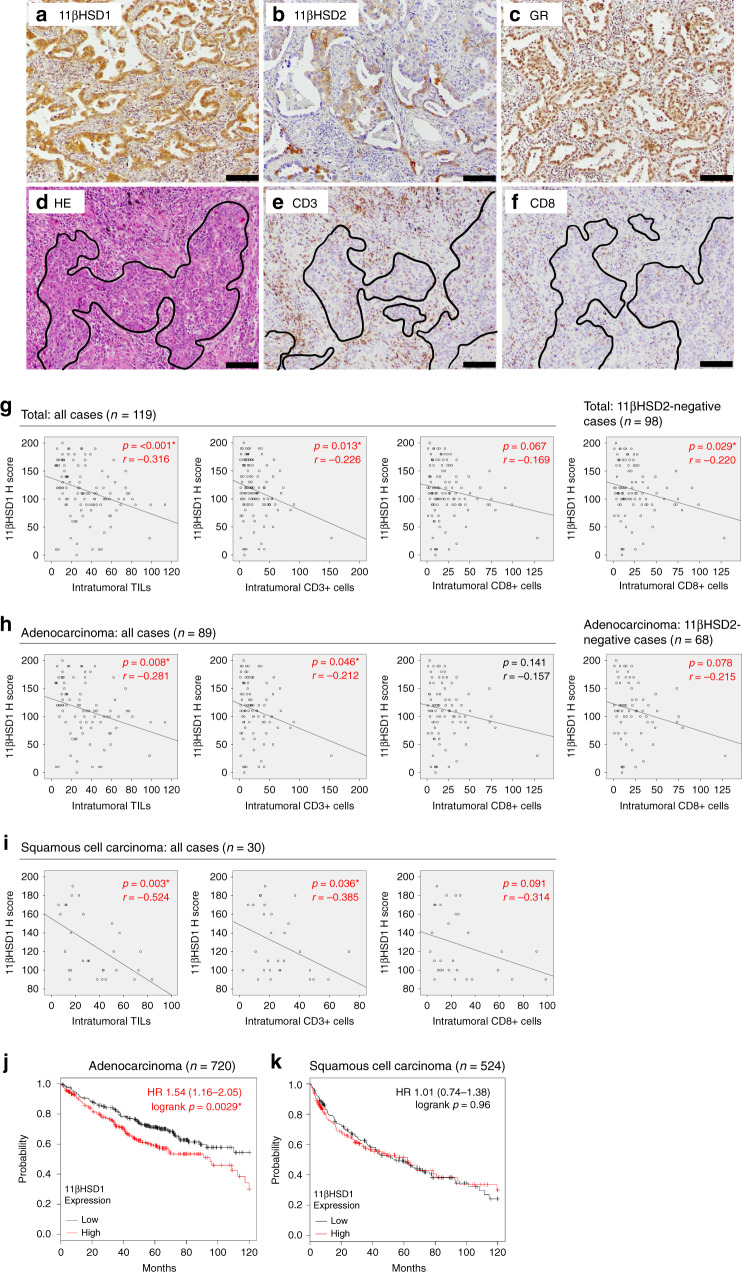
The results of in vivo analysis. **a**–**c** Representative images of 11β hydroxysteroid dehydrogenase (HSD) 1 (**a**), 11βHSD2 (**b**) and glucocorticoid receptor (GR) (**c**) immunoreactivity in non-small-cell lung carcinoma (NSCLC) specimens (bar: 100 μm). Immunoreactivity for 11βHSD1 and 2 was detected in the cytoplasm of carcinoma cells, and that for GR was detected in the nucleus of carcinoma cells. **d** Haematoxylin and eosin (HE) stain of NSCLC specimens (bar: 100 μm). Intratumoural TILs were assessed by the number of TILs infiltrating into the tumour cell nests (defined as within one tumour cell diameter from the tumour nests), which were inside of the areas surrounded by lines. **e**, **f** Immunohistochemistry for CD3 (**e**) and CD8 (**f**) (bar: 100 μm). Intratumoural CD3- or CD8-positive T cells were assessed in the same way as above. **g**–**i** The correlation between immunoreactivity of 11βHSD1 and intratumoural immune cell infiltration levels in total cases (**g**), cases with adenocarcinoma (**h**) and cases with squamous cell carcinoma (**i**). 11βHSD1 immunoreactivity was significantly and negatively correlated with intratumoural TILs and CD3- positive T cells. It tended to correlate with intratumoural CD8-positive T cells in total cases and cases with squamous cell carcinoma negatively. In 11βHSD2-negative cases, 11βHSD1 immunoreactivity in total cases was significantly associated with intratumoural CD8-positive T cells, and it tended to be associated with them in cases with adenocarcinoma. **j**, **k** The correlation between mRNA level of 11βHSD1 and overall survival in cases with adenocarcinoma (**j**) and cases with squamous cell carcinoma (**k**). The high mRNA level of 11βHSD1 was independently and significantly associated with poor overall survival of patients with adenocarcinoma. There was no significant correlation between mRNA level of 11βHSD1 and overall survival of patients with squamous cell carcinoma. *H score* histological score (modified). *p value < 0.05, significant.

Immunoreactivity was evaluated using whole tissue sections of all surgical and biopsy cases examined. Cells demonstrating higher immunointensity than the background were defined as positive cells. A modified H score was used for the assessment of 11βHSD1, 11βHSD2 and GR (Fig. 1a–c). In brief, it was obtained by adding the percentage of strongly stained cells (2×) with that of weakly stained cells (1×) in tumour cells, providing a possible range of 0–200. The amount of intratumoural immune cells was assessed by the number of these cells infiltrated into the tumour cell nests (defined as within one tumour cell diameter from the tumour nests), based on a previous report.^17^ The numbers were counted in three randomly selected fields (×400) per case, and the average of the three counts was calculated. TILs were evaluated using haematoxylin and eosin stain, whereas CD3- and CD8-positive T cells were assessed using the immunohistochemistry (Fig. 1d–f). Six cases with adenocarcinoma were not studied in the evaluation of TILs and CD3- and CD8-positive T cells, because of the presence of marked neutrophil infiltration. The evaluation was performed independently by two of the authors (R.S. and T.A.).

### Survival analysis

Survival analysis regarding 11βHSD1 in NSCLC was performed using KM-Plotter (http://www.kmplot.com/analysis/index.php?p=service&cancer=lung) at the gene expression level. KM-Plotter was an online survival analysis database to explore the prognostic value of biomarkers using transcriptomic data in various human malignancies. With this tool, the overall 10-year survival rates of 720 cases with adenocarcinoma and 524 cases with squamous cell carcinoma were analysed, respectively. 11βHSD1 was entered as the gene symbol, and the median value of 11βHSD1 expression using the JetSet probe (probe ID: 214610_at) was selected as the cut-off for the high and low 11βHSD1 groups. Multivariate Cox regression with 11βHSD1 expression and stage was performed to compute the HR and *P* values. The Kaplan–Meier and Log-Rank tests were used to estimate and display the clinical outcomes of the patients.

### Cell culture

Human cell lines used in this study included A549 (American Type Cell Culture Collection (ATCC), Manassas, VA, USA), NCI-H23 (ATCC), PC3 (Cell Resource Centre for Biomedical Research, Tohoku University, Sendai, Japan), PC9 (Riken Cell Bank, Tsukuba, Japan) and LCSC#1 (Cell Resource Centre for Biomedical Research) for lung adenocarcinoma; LK2 (Cell Resource Centre for Biomedical Research), and RERF-LC-AI (Riken Cell Bank) for lung squamous cell carcinoma; peripheral blood mononuclear cell (PBMC) from a healthy donor (Precision Bioservices, MD, USA). The lung cancer cell lines were used in previous reports from our group.^18,19^ All cells were maintained in RPMI 1640 (Sigma-Aldrich, Saint Louis, MO, USA) supplemented with 10% foetal bovine serum (FBS) (Nichirei Co. Ltd.) and 1% penicillin/streptomycin at 37 °C in a humidified incubator containing 5% CO_2_.

### Western blotting

Total protein was extracted using the PhosphoSafe Extraction Reagent (Biosciences Inc., Darmstadt, Germany) from cultured cells. Following the measurement of protein concentrations (Protein Assay Rapid Kit Wako, Wako), the total proteins were individually subjected to SDS-PAGE (SuperSep Ace, Wako). These proteins were transferred onto the Hybond P polyvinylidene difluoride membrane (GE Healthcare, Buckinghamshire, UK). Next, the proteins on the membrane were blocked in 5% non-fat dry skim milk powder (Wako) for over 1 h at room temperature and were incubated with primary antibodies overnight at 4 °C using ImmunoShot (Cosmo Bio Co., Ltd., Tokyo, Japan). The dilution of primary antibodies used in this study was as follows: 11βHSD1, 1:500; 11βHSD2, 1:1000; GR, 1:1000; β-actin (Sigma-Aldrich Co., St. Louis, MO, USA), 1:1000. These antibody–protein complexes were detected on the blot using ECL-plus western blotting detection reagents (GE Healthcare) following an incubation with anti-rabbit or anti-mouse IgG horseradish peroxidase (GE Healthcare) at room temperature for 1 h.

### Cortisol production assay

LK2 was seeded in six-well plates (5 × 10^5^ cells/ml) in the RPMI 1640 medium containing 10% FBS. Several hours later, the cells were washed with phosphate-buffered saline (PBS), and treated with 1 μM 11βHSD1 inhibitor PF915275 (Tocris/Bio-Techne, Minneapolis, USA) in phenol red- and FBS-free RPMI 1640 medium. After 2 h, the cells were incubated in phenol red- and FBS-free RPMI 1640 medium with 1 μM 11βHSD1 inhibitor PF915275 and 1 μM cortisone (Sigma Chemical Co., St. Louis, USA). After 24 h, cortisol concentration in the medium was measured using Cortisol Enzyme Immunoassay Kit (Arbor Assays, Ann Arbor, MI, USA) by Jaica (Shizuoka, Japan).

### Cytokine antibody array

As cortisol, hydrocortisone (HC) was purchased from MP Biomedicals (Solon, OH, USA). To assess the secretion levels of cytokines, we treated A549 and LK2 (1 × 10^5^ cells/ml) with ethanol as control or 100 nM HC for 24 h in the RPMI 1640 medium containing 10% FBS in six-well plates. After that, we removed the medium, washed the cells with PBS and incubated the cells in phenol red- and FBS-free RPMI 1640 medium for 24 h. The conditioned medium was used as samples. We used the Human Cytokine Antibody Array 5 (RayBiotech, Norcross, GA), which can detect 80 cytokines. The membranes were spotted with cytokine-specific antibodies and were analysed following the instructions from the manufacturer. The signal was detected using the Image Lab TM software (BIO‐RAD Laboratories, Inc., CA, USA).

### Real-time RT-PCR

Total RNA was extracted carefully from the cultured cells using the TRI reagent (Molecular Research Center, Cincinnati, OH, USA) and was reverse transcribed to cDNA using a QuantiTect Reverse Transcription Kit (Qiagen, Hilden, Germany). The levels of mRNA expression were semi-quantified using a real-time RT-PCR in a LightCycler System (Roche Diagnostics). The PCR mixture (20 μL) included 1.0 μM primer and 2× QuantiTect SYBR Green PCR Master Mix (Qiagen). The following PCR protocol was applied: initial denaturation at 95 °C for 5 min, followed by 40 amplification cycles of 95 °C for 10 s and annealing at 60 °C for 30 s. The primers used for PCR included: IL-8 forward, 5ʹ-AGGAGTGCTAAAGAACTTAGATGTCAGTGC-3ʹ; IL-8 reverse, 5ʹ-GTGGTCCACTCTCAATCACTCTCAGTTC-3ʹ; IL-6 forward, 5ʹ-TCATCTCATTCTGCGCAGCTTTAAGGAG-3ʹ; IL-6 reverse, 5ʹ-ATGCCCATTAACAACAATCTGAGGTG-3ʹ; CCL5 forward, 5ʹ-CAGTCGTCTTTGTCACCCGA-3ʹ; CCL5 reverse, 5ʹ-GAGCAAGCAGAAACAGGOAAA-3ʹ; ribosomal protein L13a (RPL13A) forward, 5ʹ-CCTGGAGGAGAAGAGGAAAG-3ʹ; RPL13A reverse, 5ʹ-TTGAGGACCTCTGTGTATTT-3ʹ. The mRNA levels of IL-8, IL-6 and CCL5 genes were expressed as ratios to the RPL13A mRNA level.

### RNA interference

The siRNA (small interference RNA) targeting GR used in this study was purchased from Sigma-Aldrich: siGR sense, 5ʹ-CCGAGAUGUUAGCUGAAAUTT-3ʹ; siGR antisense, 5ʹ-AUUUCAGCUAACAUCUCGGTT-3ʹ. siRNA 5 nM was transfected into cells (1 × 10^5^ cells/ml) using the Lipofectamine RNAi MAX reagent (Invitrogen, Carlsbad, CA, USA) for 48 h. The knockdown efficiency was assessed using immunoblotting.

### PBMC migration assay

Migration assays were performed using the Chemotaxicell containing membranes with 5-μm pore size (Kurabo, Osaka, Japan) and 24-well plates. After a treatment of LK2 (5 × 10^4^ cells/ml), which yielded the highest protein expression of GR and 11βHSD1 and the lowest expression of 11βHSD2, with 100 nM HC for 48 h in the RPMI 1640 medium containing 10% FBS, we washed the cells with PBS and exchanged the medium to phenol red- and FBS-free RPMI 1640 medium. After 24 h, we used the medium as a conditioned medium in the lower chamber. The PBMCs were plated in the upper chambers (1.5 × 10^5^ cells/well) in phenol red- and FBS-free RPMI 1640 medium. After 24 h of incubation, we counted the number of migrated cells in the lower chamber using TC 20 TM Automated Cell Counter (Bio-Rad Laboratories, Inc., CA, USA).

In order to further evaluate the cell population of migrated cells, we subsequently performed cytological examination. Migrated cells in the lower chamber were fixed in 95% ethanol or 10% formalin for 10 min, and Papanicolaou staining or immunostaining for CD3 and CD8 were performed, respectively. The ratio of the number of CD3- or CD8-positive T cells to the total cells was assessed in randomly selected three spots per microscopic field (1 mm^2^) using the software of HALO Area Quantification ver. 1.0 (Indica Laboratories, Corrales, NM).

### Statistical analysis

Statistical analysis was performed using IBM SPSS Statistics 23 (IBM Corporation, New York, USA). Comparisons between two groups of immunohistochemical analyses were performed using *t* test, *χ*^2^ tests and Pearson’s or Spearman’s correlation analysis. Statistical analyses of the in vitro study were performed using ANOVA or Tukey’s test. Statistical significance was set at *p* < 0.05 in this study.

## Results

### The correlation between the 11βHSD1, 11βHSD2 and/or GR and clinicopathological factors of the patients

Both 11βHSD1 and 11βHSD2 were detected in the cytoplasm of carcinoma cells, and GR was detected in the nuclei of these cells (Fig. 1a–c). The correlations between the immunoreactivities for 11βHSD1 or 11βHSD2 and the histological types are summarised in Table 1. 11βHSD1 H score tended to be higher in squamous cell carcinoma than adenocarcinoma (*p* = 0.063). Immunopositivity for 11βHSD2 was observed in only 21 cases of adenocarcinoma (*p* = 0.002 vs squamous cell carcinoma) with low H scores (7.4 ± 21.2). Therefore, we cautiously classified the cases into two groups: the 11βHSD2-positive (H score > 0) group and 11βHSD2-negative group (H score 0). In addition, except for only one case of squamous cell carcinoma, 124 cases demonstrated GR immunopositivity (adenocarcinoma: H score 124.2 ± 43.8, squamous cell carcinoma: H score 82.6 ± 44.5).

**Table 1:** The correlation between immunoreactivity of enzymes associated with glucocorticoid synthesis and histological type in patients with NSCLC.

	Adenocarcinoma (*n* = 95)	Squamous cell carcinoma (*n* = 30)	*p* value
11βHSD1 H score	108.2 ± 52.2	127.3 ± 34.5	0.063
11βHSD2			0.002*
Negative (H score = 0)	74	30	
Positive (H score > 0)	21 (7.4 ± 21.2)	0	

Data are presented as mean ± standard deviation.

*NSCLC* non-small-cell lung carcinoma, *11βHSD1* 11β hydroxysteroid dehydrogenase 1, *H score* histological score (modified), *11βHSD2* 11β hydroxysteroid dehydrogenase 2.

*p value < 0.05, significant.

The correlations between 11βHSD1 immunoreactivity and the examined patient’s clinical and pathological parameters are summarised in Table 2. A significant positive association was detected between 11βHSD1 immunoreactivity and age in total cases and in cases with adenocarcinoma (*p* < 0.001 and *p* = 0.001, respectively). The immunoreactivity of 11βHSD1 was significantly correlated with the smoking index, which was calculated by the number of cigarettes smoked per day multiplied by the number of years of smoking history of individuals, in cases with squamous cell carcinoma (*p* = 0.031).

**Table 2:** The correlation between 11βHSD1 immunoreactivity and clinicopathological factors in patients with NSCLC.

	Total (*n* = 125)			Adenocarcinoma (*n* = 95)			Squamous cell carcinoma (*n* = 30)		
		11βHSD1 H score or *r* (vs 11*β* HSD1 H score)	*p* value		11βHSD1 H score or *r* (vs 11*β* HSD1 H score)	*p* value		11*β* HSD1 H score or *r* (vs 114*β* HSD1 H score)	*p* value
Sex			0.718			0.953			0.513
Male	*n* = 80	114.0 ± 48.9		*n* = 53	107.9 ± 54.1		*n* = 27	125.9 ± 34.4	
Female	*n* = 45	110.6 ± 50.1		*n* = 42	108.5 ± 50.4		*n* = 3	140.0 ± 40.0	
Age	67.9 ± 8.9	0.342	<0.001*	66.9 ± 9.3	0.347	0.001*	71.3 ± 6.4	0.112	0.557
Smoking index	552.1 ± 597.3 (never smoker: *n* = 41, smoker: *n* = 84)	−0.014	0.878	424.3 ± 558.2 (never smoker: *n* = 39, smoker: *n* = 56)	−0.25	0.809	956.5 ± 541.1 (never smoker: *n* = 2, smoker: *n* = 28)	−0.394	0.031*
Stage (the 7th edition UICC TNM system for lung tumours)			0.831			0.753			0.630
I/II	*n* = 102	112.3 ± 50.8		*n* = 75	107.3 ± 54.7		*n* = 27	126.3 ± 34.4	
III/IV	*n* = 23	114.7 ± 41.9		*n* = 20	111.5 ± 42.0		*n* = 3	136.6 ± 41.6	
EGFR mutation						0.552			
Negative				*n* = 46	111.5 ± 56.1				
Positive				*n* = 49	105.1 ± 48.7				

Data are presented as mean ± standard deviation.

*11βHSD1* 11β hydroxysteroid dehydrogenase 1, *NSCLC* non-small-cell lung carcinoma, *H score* histological score (modified), *UICC* Union for International Cancer Control, *EGFR* epidermal growth factor receptor.

*p value < 0.05, significant.

### The correlation between 11βHSD1 or GR and the infiltration of immune cells in tumour tissues

The results regarding the infiltration level of immune cells are shown in Fig. 1d–i. The immunoreactivity of 11βHSD1 was inversely correlated with intratumoural TILs and CD3-positive T cells [(*p* < 0.001, *r* = −0.316 and *p* = 0.013, *r* = −0.226 in all cases (Fig. 1g); *p* = 0.008, *r* = −0.281 and *p* = 0.046, *r* = −0.212 in cases with adenocarcinoma (Fig. 1h); *p* = 0.003, *r* = −0.525 and *p* = 0.036, *r* = −0.385 in cases with squamous cell carcinoma, respectively (Fig. 1i)]. We observed a trend of inverse correlation between 11βHSD1 immunoreactivity and intratumoural CD8-positive T cells in all cases and in cases with squamous cell carcinoma (*p* = 0.067, *r* = −0.169 and *p* = 0.091, *r* = −0.314, respectively) (Fig. 1g, i). In 11βHSD2-negative cases, 11βHSD1 immunoreactivity in all cases was significantly associated with intratumoural CD8-positive T cells (*p* = 0.029, *r* = −0.220) (Fig. 1g), and a similar trend was observed in cases of adenocarcinoma (*p* = 0.078, *r* = −0.215) (Fig. 1h). There was no significant association between GR immunoreactivity and the infiltration level of TILs, CD3- or CD8-positive T cells (*p* = 0.600, *p* = 0.796 and *p* = 0.938 in all cases; *p* = 0.843, *p* = 0.907 and *p* = 0.557 in cases with adenocarcinoma; *p* = 0.511, *p* = 0.321 and *p* = 0.896 in cases with squamous cell carcinoma, respectively).

### The correlation between 11βHSD1 status and the efficacy of IC blockade therapy

Clinicopathological characteristics of the cases examined are summarised in Table 3. The correlation between 11βHSD1 immunoreactivity in carcinoma cells and therapeutic efficacy is summarised in Table 4. The cases were tentatively classified into low and high 11βHSD1- expression groups. We applied the average of 11βHSD1 H score (119.4) to the cut-off of these two groups. High 11βHSD1-expression group had significantly higher progressive disease than low 11βHSD1-expression group (*p* = 0.038). In addition, the progressive disease group tended to harbour a higher 11βHSD1 H score than the partial remission group, but the difference did not reach statistical significance (*p* = 0.059).

**Table 3:** The clinicopathological characteristics of examined cases.

Case	Sex	Age	Smoking index	Stage (the 7th edition UICC TNM system for lung tumours)	Histology	Specimen	Treatment history	Therapeutic efficacy (the RECIST version 1.1)	Number of courses of pembrolizumab at the time of assessment of therapeutic efficacy
1	M	69	1000	3	Adenocarcinoma	Biopsy	Pembrolizumab	PR	4
2	M	63	800	4	Adenocarcinoma	Biopsy	Pembrolizumab	PR	3
3	F	59	600	4	Adenocarcinoma	Biopsy	Pembrolizumab	PD	2
4	M	53	1170	4	Adenocarcinoma	Biopsy	Pembrolizumab	PR	3
5	M	53	800	4	Adenocarcinoma	Biopsy	Pembrolizumab	PR	3
6	F	76	120	3	Adenocarcinoma	Biopsy	Pembrolizumab	PR	4
7	M	53	390	4	Adenocarcinoma	Biopsy	Pembrolizumab	PR	4
8	M	74	108	4	Squamous cell carcinoma	Biopsy	Pembrolizumab	PR	4
9	F	72	0	4	Non-small-cell carcinoma	Biopsy	Pembrolizumab	PD	3
10	M	67	600	4	Non-small-cell carcinoma	Biopsy	Pembrolizumab	PD	4
11	M	75	1500	3	Adenocarcinoma	Biopsy	Pembrolizumab + CBDCA + PEM	PR	9
12	M	79	2500	4	Adenocarcinoma	Biopsy	Pembrolizumab + CBDCA + PEM	PR	4
13	M	74	1080	3	Adenocarcinoma	Biopsy	Pembrolizumab + CBDCA + PTX	PR	2
14	F	49	0	4	Adenocarcinoma	Biopsy	Pembrolizumab + CDDP + PEM	PD	3
15	M	63	1600	4	Non-small-cell carcinoma	Biopsy	Pembrolizumab + γknife	PD	6
16	M	69	1200	2	Adenocarcinoma	Surgery	Surgical resection of primary lesion → Recurrence (7 months after the surgery) → Pembrolizumab	PR	2
17	M	56	1280	3	Adenocarcinoma	Biopsy	Surgical resection of primary lesion and adjuvant chemotherapy → Recurrence (6 months after the surgery) biopsy performed → Pembrolizumab	PD	11
18	M	63	400	2	Pleomorphic carcinoma	Surgery	Surgical resection of primary lesion → Recurrence (several days after the surgery) → Pembrolizumab	PR	4

*UICC* Union for International Cancer Control, *RECIST* Response Evaluation Criteria in Solid Tumours, *M* male, *F* female, *CBDCA* carboplatin, *PEM* pemetrexed, *PTX* paclitaxel, *CDDP* cisplatin, *PR* partial remission, *PD* progressive disease.

**Table 4:** The correlation between immunoreactivity of 11βHSD1 and therapeutic efficacy of IC blockade therapy in NSCLC.

	11βHSD1 H score (low/high)			11βHSD1 H score	
	Low ≦ 119 (*n* = 7)	High ≧ 120 (*n* = 11)	*p* value		*p* value
PR (*n* = 12)	7	5	0.038*	101.6 ± 60.7	0.059
PD (*n* = 6)	0	6		155.0 ± 25.8	

Data are presented as mean ± standard deviation.

*11βHSD1* 11β hydroxysteroid dehydrogenase 1, *NSCLC* non-small-cell lung carcinoma, *H score* histological score (modified), *PR* partial remission, *PD* progressive disease.

*p value <0.05, significant.

### The correlation between the mRNA levels of 11βHSD1 and the overall survival of the patients

As summarised in Fig. 1j, a high gene expression level of 11βHSD1 was independently and significantly associated with poor overall survival of patients with adenocarcinoma (univariate analysis: HR 1.54 [1.16–2.05], *p* = 0.0029) (multivariate analysis: 11βHSD1: HR 1.38 [1.03–1.85], *p* = 0.0306, Stage: HR 1.79 [1.49–2.15], *p* < 0.001). There was no significant correlation between gene expression levels of 11βHSD1 and the overall survival of patients with squamous cell carcinoma (Univariate analysis: HR 1.01 [0.74–1.38], *p* = 0.96) (Multivariate analysis: 11βHSD1: HR 1.38 [1.03–1.85], *p* = 0.8276, Stage: HR 1.32 [1.07–1.62], *p* = 0.0083) (Fig. 1k).

### The expression of GC synthesis-associated enzymes and receptors in NSCLC cell lines

**Fig. 2:**
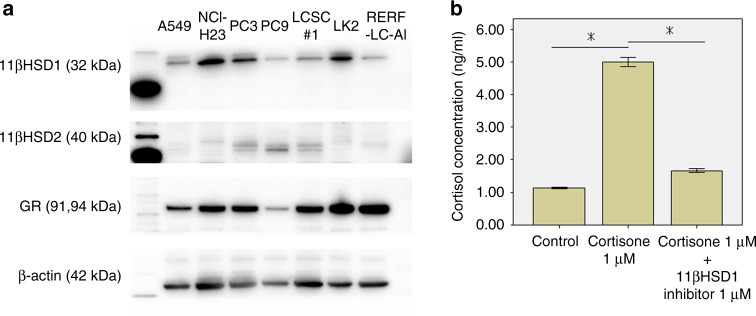
The status of cortisol synthesis. **a** The expression of glucocorticoid synthesis-associated enzyme and receptor in non-small-cell lung carcinoma (NSCLC) cell lines. Most NSCLC cell lines other than PC9 demonstrated higher protein expression of 11β hydroxysteroid dehydrogenase (HSD) 1 than 11βHSD2, and high expression of glucocorticoid receptor (GR). **b** Results of cortisol synthesis assay. Cortisol concentration was significantly increased by 1 µM cortisone disposure for 24 h. This increase was significantly suppressed by 1 µM 11βHSD1 inhibitor treatment. Values are mean ± standard deviation (*n* = 3). *p value < 0.05, significant.

NSCLC cell lines other than PC9 had relatively abundant levels of 11βHSD1 protein expression compared with 11βHSD2 and had high expression of GR (Fig. 2a).

### Induction of cortisol synthesis through 11βHSD1 in NSCLC cells

The results of a cortisol synthesis assay using LK2 are shown in Fig. 2b. Cortisol concentration was significantly increased by 1 µM cortisone disposure for 24 h (*p* < 0.001). This increase was significantly suppressed by 1 µM 11βHSD1 inhibitor treatment (*p* < 0.001).

### The effect of GC on expression of cytokines in NSCLC cells

**Fig. 3:**
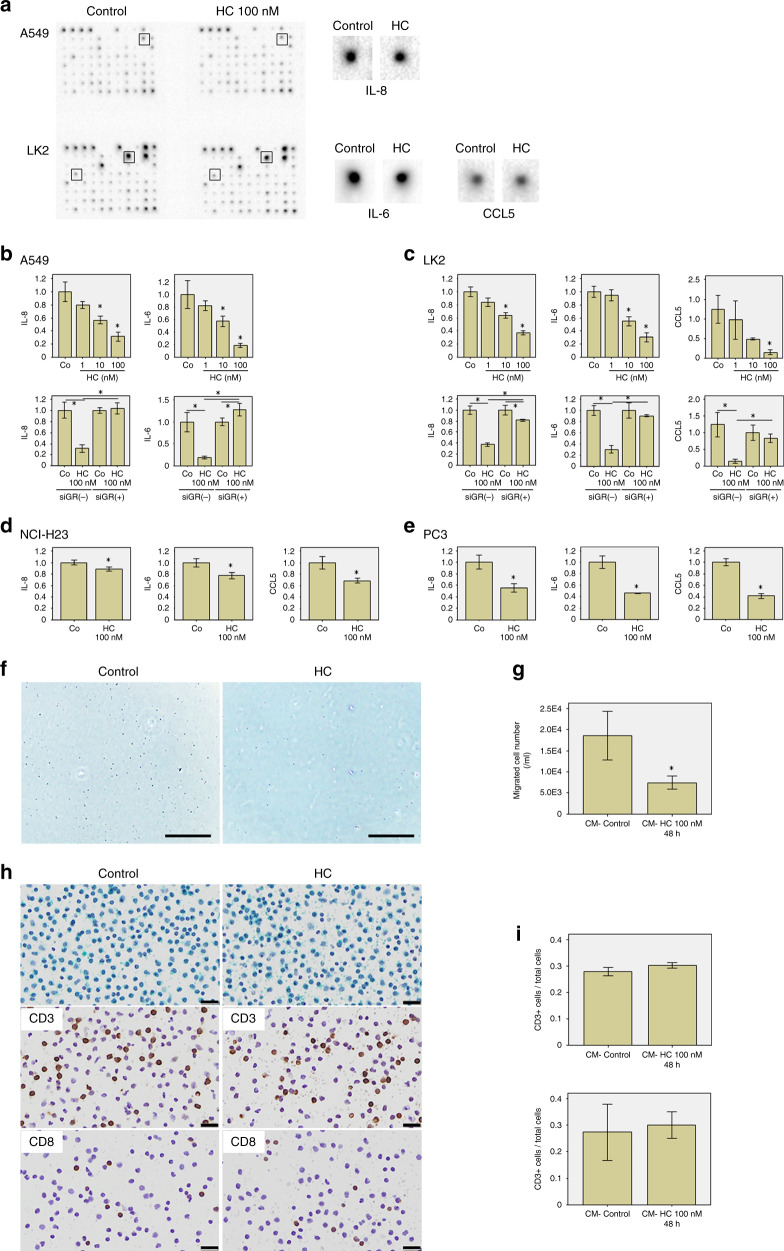
The effects of cortisol on cytokine production and immune cell migration. **a** A result of cytokine antibody array. Especially, among cytokines associating with lymphocyte migration, interleukin (IL)-8, IL-6 and chemokine (C–C motif) ligand 5 (CCL5) protein expression appeared to be slightly reduced by exposure to hydrocortisone (HC) [100 nM, 24 h, in RPMI 1640 medium containing 10% foetal bovine serum (FBS)]. **b**–**e** The effect of HC on mRNA expression of IL-8, IL-6 and CCL5 in A549 (**b**), LK2 (**c**), NCI-H23 (**d**) and PC3 (**e**). Data were calculated by fold change versus control (Co). mRNA expression of IL-8 and IL-6 was significantly reduced by HC treatment in four cell lines, and that of CCL5 was significantly reduced in three cell lines (100 nM, 48 h, in RPMI 1640 medium containing 10% FBS for A549 and LK2, in charcoal-stripped RPMI 1640 medium containing 10% charcoal-stripped FBS for NCI-H23 and PC3). Dose dependency was confirmed in A549 and LK2. Glucocorticoid receptor (GR) dependency was confirmed by knockdown of GR using siGR in A549 and LK2. **f**–**i** Peripheral blood mononuclear cell (PBMC) migration assay. **f** The images of the lower chamber using conditioned medium (CM)—control and HC (100 nM) (bar: 200 μm). **g** Treatment by HC (100 nM, 48 h RPMI 1640 medium containing FBS) inhibited the migration ability of PBMC in LK2. **h**, **i** Assessment of the subpopulation of migrated cells using Papanicolaou staining and immunostaining for CD3 and CD8. **h** Representative images of Papanicolaou staining and immunocytochemistry for CD3 and CD8. **i** HC treatment did not alter the ratio of the number of CD3- or CD8-positive T cells to the total migrated cells. Values are mean ± standard deviation (*n* = 3). *p value < 0.05, significant.

The cytokine antibody array revealed that among the cytokines associated with lymphocyte migration, IL-8, IL-6, CCL5, (C–X–C motif) ligand (CXCL) 5, CXCL10, IL-10, transforming growth factor β and vascular endothelial growth factor protein expression appeared to be changed by the exposure to HC (100 nM, 24 h, in RPMI 1640 medium containing 10% FBS) (Fig. 3a). We validated the mRNA expression levels of these cytokines and did confirm that those of IL-8 and IL-6 were significantly reduced by the HC treatment (100 nM, 48 h, in RPMI 1640 medium containing 10% FBS for A549 and LK2, in charcoal-stripped RPMI 1640 medium containing 10% charcoal-stripped FBS for H23 and PC3) in four cell lines (A549: *p* < 0.001 and *p* < 0.001, LK2: *p* < 0.001 and *p* < 0.001, H23: *p* = 0.032 and *p* = 0.014 and PC3: *p* = 0.020 and *p* = 0.007, respectively), and those of CCL5 in three cell lines other than A549 (LK2: *p* = 0.010, NCI-H23: *p* = 0.010 and PC3: *p* = 0.002, respectively) (Fig. 3b–e). A dose dependency was confirmed in A549 (IL-8: *p* = 0.002 [HC 10 nM vs control], *p* = 0.035 [HC 100 nM vs 10 nM]; IL-6: *p* = 0.014 [HC 10 nM vs control], *p* = 0.024 [HC 100 nM vs 10 nM]) and LK2 (IL-8: *p* < 0.001 [HC 10 nM vs control], *p* = 0.002 [HC 100 nM vs 10 nM]; IL-6: *p* < 0.001 [HC 10 nM vs control], *p* = 0.019 [HC 100 nM vs 10 nM]; CCL5: *p* = 0.061 [HC 10 nM vs control], *p* = 0.010 [HC 100 nM vs 10 nM]) (Fig. 3b, c). Furthermore, a GR dependency was confirmed by the knockdown of GR using the siGR in A549 (IL-8: *p* = 0.540 [siGR (+) control vs siGR (+) HC 100 nM], *p* = 0.001 [siGR (–) HC 100 nM vs siGR (+) HC 100 nM]; IL-6: *p* = 0.039 [siGR (+) control vs siGR (+) HC 100 nM], *p* < 0.001 [siGR (–) HC 100 nM vs siGR (+) HC 100 nM]) and LK2 (IL-8: *p* = 0.0.22 [siGR (+) control vs siGR (+) HC 100 nM], *p* < 0.001 [siGR (–) HC 100 nM vs siGR (+) HC 100 nM]; IL-6: *p* = 0.323 [siGR (+) control vs siGR (+) HC 100 nM], *p* < 0.001 [siGR (–) HC 100 nM vs siGR (+) HC 100 nM]; CCL5: *p* = 0.332 [siGR (+) control vs siGR (+) HC 100 nM], *p* = 0.006 [siGR (–) HC 100 nM vs siGR (+) HC 100 nM]) (Fig. 3b, c).

### The effect of GC on the migration ability of PBMC

The HC treatment (100 nM, 48 h in RPMI 1640 medium  containing FBS) significantly inhibited the migration ability of PBMC in LK2 (*p* = 0.032) (Fig. 3f, g). The migrated cells consisted of lymphocytes, monocytes and a few granulocytes in the light microscopic examination on Papanicolaou stained slides (Fig. 3h). HC treatment did not alter the ratio of the number of CD3- and CD8-positive T cells to the total migrated cells (*p* = 0.101, *p* = 0.714, respectively) (Fig. 3h, i).

## Discussion

In this study, we initially demonstrated that 11βHSD1 was involved in the in situ activation of GC, and was significantly and inversely correlated with the number of TILs and T cells in NSCLC tissues. In addition, cortisol was shown to reduce the expression of cytokines, such as CCL5, IL-8 and IL-6, resulting in the inhibition of migration of monocyte, including T cells and CD8-positive T cells without changing their population. All of these results indicated that cortisol exerted inhibitory effects on the antitumour immune response via the inhibition of T-cell migration into the tumours of NSCLC.

This is also the first report to clarify the significance of intratumoural GC synthesis in NSCLC, as this predicts the efficacy of IC blockade therapy and the clinical outcome of patients. The tissue concentration of cortisol, synthesised intratumourally in the de novo pathway, has not been reported previously. However, it was reported that the intratumoural concentration of sex steroids in tumours harbouring high expression levels of synthesising enzymes, was significantly higher than that in normal tissues.^20,21^ In addition, according to our in vitro study as shown in Fig. 2b, significant increase in intratumoural 11βHSD1-dependent cortisol production in 11βHSD1-expressing NSCLC cells was confirmed. Therefore, the tumours demonstrating relatively abundant expression of 11βHSD1 are reasonably assumed to produce glucocorticoid. This results in a high intratumoural cortisol concentration. However, these findings await further investigation, such as direct measurement of intratumoural levels of cortisol in NSCLC.

The level of 11βHSD1 was inversely correlated with the amount of infiltrating immune cells in the tumour as shown in Fig. 1g–i. These results suggested that intratumourally synthesised biologically active GC cortisol, converted by 11βHSD1 in tumour cells, could inhibit the immune response in areas adjacent to the tumour cells via GR activation, in an autocrine fashion. We considered that this inhibitory effect on the infiltration of CD8-positive T cells was reduced in the presence of 11βHSD2, which was involved in the inactivation of intratumourally synthesised cortisol. This hypothesis was supported by the fact that the correlation between 11βHSD1 immunoreactivity in tumour cells and intratumoural CD8-positive T cells was more marked in 11βHSD2-negative cases among total cases as well as adenocarcinoma cases (Fig. 1g, h). GR immunoreactivity in tumour cells was not significantly associated with the amount of infiltrating immune cells in the tumour, although the correlation between GR status and the intratumoural immune microenvironment has not been examined in any human malignancy. Therefore, the presence of ligand is more important than GR expression for the detection of GR action on the intratumoural immune microenvironment. In addition, it is well-known that the infiltration of immune cells, especially the intratumoural CD8-positive T cells, is an important predictor of the effective response to IC blockade therapy.^3,4^ Accordingly, it is considered that 11βHSD1 is a new indicator for poor response to IC blockade therapy, and the additional use of 11βHSD1 inhibitor may increase the efficacy of IC blockade therapy in NSCLC. This hypothesis was supported by the results of our present study that a significantly inverse correlation was detected between 11βHSD1 immunoreactivity in tumour cells and the efficacy of IC blockade therapy (Table 4). However, it is also true that we examined only 18 cases. Therefore, the predictive value of 11βHSD1 on the efficacy of IC blockade therapy has not necessarily been established and it awaits further investigations for clarification.

Furthermore, some previous reports have stated that immune cell infiltration seemed to suppress tumour cells and improve the long-term survival in several tumour types, including NSCLC, small-cell lung cancer, breast cancer and renal-cell carcinoma.^22–26^ Based on these reports and our immunohistochemical analysis, it was assumed that the intratumoural levels of 11βHSD1 could predict poor prognosis of NSCLC. Of particular interest are the results of our prognostic analysis using the KM plotter. These results were also consistent with these predictive findings in lung adenocarcinoma.

We performed in vitro studies in order to validate the immunohistochemical results and to further clarify the mechanism of cortisol effects on the intratumoural immune microenvironment. The results revealed that active GC suppressed the migration of immune cells via the inhibition of cytokines expression such as CCL5, IL-8 and IL-6 as shown in Fig. 3. CCL5 is a well-known chemotactic cytokine for T cells, monocytes and others, and CCL5 has been reported to attract CD3- and CD8-positive T cells into various tumours as well as NSCLC resulting in good outcome.^27,28^ Therefore, the results of our present study did support our hypothesis based on those of immunohistochemical analyses in clinical materials, i.e., an association of 11βHSD1 in tumour cells with the inhibition of recruitment of CD3- and CD8-positive T cells. The well-known chemokine IL-8, induces the migration of neutrophils and lymphocytes. IL-8 acts directly or indirectly on the immune cells through the regulation of other cytokines such as IL-2 and IL-10.^29,30^ Previous studies have reported an association between IL-8 and T-cell induction in several types of human malignancies. This includes malignant melanoma, oesophagus adenocarcinoma, ovarian cancer and so on.^31–33^ Several reports describe the effect of IL-6 on intratumoural T-cell induction.^34,35^ However, IL-6 is a multifunctional cytokine with both immunosuppressive and immune-promotive effects.^36,37^ Further investigation is required regarding the clarification of its actions in this area. Although our study focused on the effects of GC on GR in tumour cells, it was shown that GC could directly act on the GR of lymphocytes. This resulted in the inhibition of proliferation, and T-cell receptor signalling, as well as the induction of apoptosis in lymphocytes.^38,39^ Therefore, we must consider the possibility that the intratumourally produced GC reduced T-cell infiltration via the induction of lymphocyte apoptosis. Further investigation is required to elucidate the complex mechanisms of the inhibitory effect of GC on immune responses.

In conclusion, we initially demonstrated the significant negative impact of cortisol, including that intratumourally synthesised through 11βHSD1, on the intratumoural immune microenvironment in NSCLC. It is also true that further studies, such as tumour-killing assays or experiments using animal models of spontaneous lung cancer, are required for clarification, but the results of our present study could provide new insights into therapeutic strategies, and these results may also improve the predictive accuracy of outcomes in IC blockade therapy.

## Data Availability

All data generated or analysed during this study are included in this published article.
